# Evaluating Sleep Challenges in Hospitalized Youth

**DOI:** 10.7759/cureus.63302

**Published:** 2024-06-27

**Authors:** Abigail R Strang, Daniela Uribe, David Rappaport, Seema Rani, Aaron Chidekel

**Affiliations:** 1 Department of Pediatrics, Division of Pediatric Pulmonology, Sidney Kimmel Medical College of Thomas Jefferson University/Nemours Children's Health, Wilmington, USA; 2 Department of Pediatrics, Division of General Academic Pediatrics, Sidney Kimmel Medical College of Thomas Jefferson University/Nemours Children's Health, Wilmington, USA

**Keywords:** quality improvement, mental health, vital signs, pediatrics, sleep disruption

## Abstract

Objective: To characterize sleep quality and sleep disruptions among youth hospitalized outside of the intensive care unit (ICU).

Patients and methods: Participants were eligible for the survey-based study if they were 8-17 years old, English-speaking, hospitalized for ≥3 days outside of the ICU, and developmentally able to understand surveys. Survey administration included a sleep diary, the Epworth Sleepiness Scale for Children and Adolescents (ESS-CHAD), and a study-specific Inpatient Sleep Disruptors Questionnaire. The chart review provided additional clinical information. Descriptive and comparative statistics were performed to assess the association between overnight clinical monitoring and daytime sleepiness.

Results: Forty-five participants (mean age 13.4 years, 60% female), recruited between May and December 2022, were included in the study. Mean total sleep time (8.2 ± 1.7 hours) and ESS-CHAD score (8.6 ± 4.3) were normal with 79% reporting fair to good sleep the previous night. Participants rated alarms on equipment, vital signs, and noise as most disruptive to sleep. Participants with vital signs every four hours showed higher levels of daytime sleepiness compared with participants with vitals measured every shift (9.3 vs. 6.3; p=0.04).

Conclusions: Most participants reported normal sleep, although there was wide variability with a portion with impaired sleep quality and elevated daytime sleepiness. Alarms on equipment, vital signs, and noise were most disruptive, and increased vital sign frequency was also associated with increased daytime sleepiness. In clinically stable pediatric patients, a reduction in vital sign monitoring overnight may be an important change to improve patient sleep.

## Introduction

Sleep in children and adolescents plays a crucial role in general health and wellness, especially during times of illness and stress. Pediatric sleep health encompasses a comprehensive assessment of individual and family satisfaction with sleep, appropriate sleep timing and duration, sleep efficiency, healthy sleep behaviors, and daytime alertness [1]. Impaired sleep health in children has a wide range of negative health effects including effects on the immune, respiratory, cardiovascular, metabolic, and neurocognitive systems [2]. Despite the well-established need for adequate sleep for patients recovering from injury or illness, hospitalized patients are at high risk for poor sleep [3]. Insufficient sleep during recovery from illness and injury has been associated with adverse health outcomes in adults, even increased mortality [4]. Prior studies have demonstrated that pain and overnight interruptions are the most important factors impacting sleep quality during pediatric hospitalizations [5]. Compared with adults, factors affecting sleep quality in children are not as well-studied, especially outside of intensive care unit (ICU) settings. Studies in pediatrics have found that patients obtain less than the recommended amounts of sleep for their age and have high levels of night-time traffic into their hospital rooms, often causing sleep disruption [3]. In one large study of pediatric patients, factors reported as most disruptive to sleep were vital signs, physician/nurse interruptions, and pulse oximetry [6].

Poor sleep in the hospital may also establish poor sleep habits, which may continue after discharge and affect recovery at home [7]. Patients with long hospital stays have elevated amounts of daytime sleep, which can have lasting negative implications for overall health and recovery [8]. Therefore, it is important for the medical team to prioritize sleep during hospitalization and develop systems and environments that are conducive to high-quality sleep. An increased understanding of sleep quality and factors that disrupt sleep in pediatric patients is therefore imperative.

The primary aim of this study was to characterize patient-reported sleep, daytime sleepiness, and factors causing sleep disruption among hospitalized pediatric patients outside of the ICU. Additionally, we explored the association between daytime sleepiness and the frequency of overnight clinical monitoring. 

## Materials and methods

This survey-based study was approved by the hospital’s Institutional Review Board. Eligible participants in this convenience sample were recruited when a research team member was available (typically Monday-Friday, 8 AM-5 PM) and when the participant was available (times without medical treatment, discussions with medical team/rounding, or testing/procedures). Research team members were not involved in the care of the patient. Recruitment occurred between May 2022 and December 2022.

Participants were eligible if they were between the ages of eight and 17 years, hospitalized outside of the ICU, and hospitalized for ≥3 calendar days. We included patients only after ≥3 calendar days to capture patients who were not within 24 hours of an emergency room visit or surgical procedure and those hospitalized longer than a typical observation period. We included patients who were hospitalized primarily for a medical diagnosis or rehabilitation after surgery rather than a primary behavioral health diagnosis. Additionally, participants were required to be English-speaking (as not all tools were available in non-English languages) and developmentally able to understand the surveys with assistance from a parent or the research team member. Eligible participants were identified via chart review and approached in-person by a member of the research team to discuss enrollment. After caregiver consent and participant assent were completed, survey administration was completed in a single study visit, typically in about 20 minutes. No specific incentive or reward was offered for participation.

After the research visit, the research team performed a chart review to obtain additional clinical and demographic data. Specifically, the research team reviewed the following data: age, sex at birth, race, ethnicity, admission diagnosis, primary admission team (general pediatrics, pulmonology, rehabilitation medicine, hematology/oncology, gastroenterology), day of hospitalization, diagnosis of sleep disorder (including insomnia, sleep apnea, or other documented sleep disorder), prescription of sleep aid (melatonin or other sedative/hypnotic), use of pulse oximetry overnight (either continuous or with vital signs), and frequency of vital sign monitoring (every four hours or every eight-hour nursing shift).

Comprehensive aspects of sleep health were measured through the administration of three survey-based tools. To measure participant-reported sleep times, perceived awakenings, and quality of sleep, we used a detailed sleep diary (adapted from the Consensus Sleep Diary) [9]. Participants were asked to estimate sleep times and quantity, number of awakenings, and rate the quality of their sleep, either 0 (very poor), 1 (poor), 2 (fair), 3 (good), and 4 (very good). Participants were asked to estimate timings and awakenings based on memory of the prior night’s sleep to the best of their ability.

Daytime sleepiness was measured using the Epworth Sleepiness Scale for Children and Adolescents (ESS-CHAD) [10]. This tool is an eight-item, validated survey for assessing daytime sleepiness across different settings in children with a total score of 0 to 24 (higher scores = higher levels of daytime sleepiness).

The Inpatient Sleep Disruptors Questionnaire was adapted from a similar hospital-based survey [5]. Patients were presented with 15 potential sleep disruptors including five medical disruptors (vital signs, medical staff in room, medications, blood draw, other medical procedure), two emotional disruptors (feeling anxious, pain), and eight environmental disruptors (all noise, bed comfort, staff conversations, alarms on equipment, room temperature, television in room, cell phone or tablet in room). Patients were asked to rank these disruptors 1-5 in terms of their importance in affecting their previous night of sleep, with 1 being least disruptive and 5 being most disruptive.

Descriptive analytical statistics were used for demographic information, clinical characteristics, and survey results as appropriate. For continuous variables, the mean and standard deviation were calculated. For categorical variables, percentages of participants in each category were calculated. Selected questions from the Inpatient Sleep Disruptors Questionnaire and the Consensus Sleep Diary were examined on the item level. Excessive daytime sleepiness was measured using the ESS-CHAD and the association of these results with the type/frequency of clinical monitoring. Unpaired t-tests were used to compare participants with elevated (≥11) ESS-CHAD scores with those with normal scores (≤10) based on clinical monitoring: pulse oximetry and vital signs. Continuous pulse oximetry was compared with pulse oximetry measured every four hours, and vital signs taken every four hours were compared with vital signs taken every eight-hour nursing shift.

## Results

Overall, 60 potential participants were approached by a member of the research team, and 45 participants (60% female, 58% White, mean age 13.4 ± 2.6 years) completed the study. The study visit occurred, on average, on day 8.5 ± 12 of the hospitalization. Participants were recruited from various teams including general pediatrics (n=16; 36%), pediatric rehabilitation medicine (n=11; 24%), hematology/oncology (n=11; 24%), pulmonology (n=3; 7%), and gastroenterology (n=4; 9%). Out of the potential participants who were approached but did not participate in the study, the most common reason for not participating was cognitively or developmentally unable to understand the surveys at the time of survey administration. As shown in Table 1, there was a wide variety of primary admission diagnoses, most commonly “infection requiring intravenous antibiotics” (n=7, 16%). Most of the participants in this study (n=36, 80%) did not have a diagnosed sleep disorder and were not prescribed one or more sleep aids during the hospitalization (n=38; 84%). Relatively few participants had a diagnosis of sleep apnea (n=5; 11%) or insomnia (n=4; 9%).

**Table 1:** Participant characteristics

Characteristic	Mean, standard deviation/n (%)
Age, mean, standard deviation	13.4, 2.6
Sex, n (%)	
Male	18 (40)
Female	27 (60)
Days since admission, mean, standard deviation	8.5, 12
Primary admission diagnosis, n (%)	
Postoperative after orthopedic surgery	6 (13)
Infection needing intravenous antibiotics	7 (16)
Primary gastrointestinal problem	6 (13)
Pain crisis	6 (13)
Traumatic brain injury	5 (11)
Other	15 (33)
Primary inpatient team, n (%)	
Pulmonology	3 (7)
Rehabilitation	11 (24)
General pediatrics	16 (36)
Gastroenterology	4 (9)
Hematology/oncology	11 (24)
Diagnosed sleep disorder, n (%)	
Sleep apnea	5 (11)
Insomnia	4 (9)
None	36 (80)
Sleep aid prescribed, n (%)	
Yes	7 (16)
No	38 (84)
Vital signs order, n (%)	
Every shift	12 (27)
Every 4 hours	33 (73)
Use of continuous pulse oximetry, n (%)	
Yes	16 (36)
No	29 (64)

Sleep Times, Quality, and Sleepiness

The mean reported total sleep time for participants was 8.2 ± 1.7 hours (range 5-12 hours). The mean number of nocturnal awakenings was 2.2 (range 0-7). Six (14%) participants rated their sleep quality as “poor” or “very poor,” while 17 (39%) reported “fair” sleep quality. Twenty-one participants (48%) reported “good” or “very good” sleep quality.

The average ESS-CHAD score for the entire cohort was 8.6 ± 4.3 with a wide range of 1-24. The majority (73%) of participants reported normal levels of daytime sleepiness. Of the 27% of participants with increased daytime sleepiness, four (9%) reported mild excessive daytime sleepiness, six (14%) reported moderate excessive daytime sleepiness, and two (5%) reported severe excessive daytime sleepiness. See Table 2 for full survey results.

**Table 2:** Survey results

Variable	Mean (standard deviation)/n (%)/Mean (range)
Epworth Sleepiness Scale (n=44)	
Mean (standard deviation)	8.6 (4.3)
Categorical scoring for excessive daytime sleepiness, n (%)	
Lower normal	11 (25)
Higher normal	21 (48)
Mild excessive	4 (9)
Moderate excessive	6 (14)
Severe excessive	2 (5)
Consensus sleep diary (n=45)	
Total sleep time, mean (range)	8.2 (5–12)
Nighttime awakenings, mean (range)	2.2 (0–7)
How do you rate the quality of your sleep? (n=44), n (%)	
Very poor	2 (5)
Poor	4 (9)
Fair	17 (39)
Good	17 (39)
Very good	4 (9)
How rested or refreshed did you feel when you woke up for the day? (n=42), n (%)	
Not at all rested	4 (10)
Slightly rested	8 (19)
Somewhat rested	15 (36)
Well-rested	13 (31)
Very well-rested	2 (5)

Sleep Disruptors

**Figure 1:**
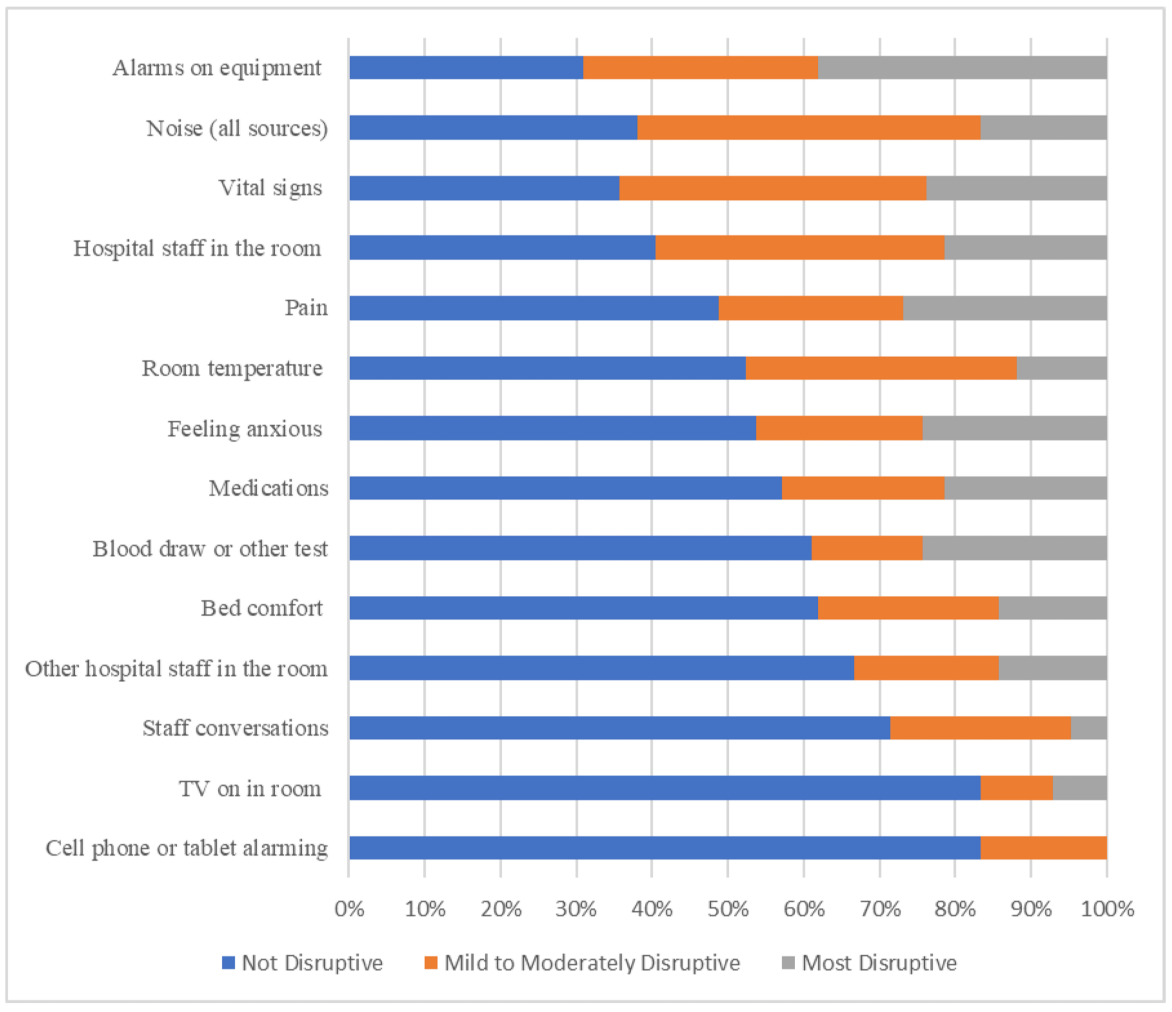
Results from the Inpatient Sleep Disruptors Survey

On the Inpatient Sleep Disruptors Questionnaire, the factors that caused the most sleep disruption (rated as at least mildly disruptive) were alarms on equipment (69%), vital signs (64%), noise (63%), and hospital staff (60%). Patients were least disrupted by cell phones, tablets, or televisions in the room; 83% of patients reported “not being disrupted at all” by these two factors. See Figure 1 for the full results of the survey.

Clinical Monitoring

Most patients had vital signs measured every four hours overnight (73%), while the remainder were measured every eight-hour nursing shift. Overnight continuous pulse oximetry was utilized in 36% of participants. Additional analysis was performed to evaluate for any differences in sleepiness based on the frequency of clinical monitoring (vital signs and pulse oximetry). Patients with vital signs measured every four hours had a higher (worse) score on ESS-CHAD compared with patients monitored every eight-hour nursing shift (9.3 vs. 6.3; p=0.04). In contrast, there was no statistically significant difference in ESS-CHAD scores associated with the use of continuous pulse oximetry overnight compared with spot-check pulse oximetry (8.9 vs. 8.4; p=0.7).

## Discussion

This survey-based study represents an assessment of overall sleep health in hospitalized children outside of the ICU from the patient and family’s perspective. Somewhat surprisingly, most participants reported overall normal amounts of sleep and fair to good sleep quality based on subjective measures. Levels of daytime sleepiness were also typically normal, although a portion of participants did report elevated daytime sleepiness and poor-quality sleep. The most disruptive factors to patients’ sleep overnight were hospital-based environmental factors, including alarms on equipment, vital sign measurements, noise, and hospital staff, with the least disruptive being personal technology such as cell phones and television. The factors that the participants ranked as highly disruptive (vital signs) also correlated with another measure of impaired sleep health, increased daytime sleepiness.

Novel findings in this study include the description of overall sleep quality and sleep disruptors in pediatric patients outside of the ICU setting and primarily from the patient and caregiver perspective. While other studies have evaluated sleep disruptors in adult patients and pediatric patients in ICU settings [2], this study assesses overall pediatric sleep in children who are not critically ill. In comparison to children who are hospitalized in an ICU setting and have high levels of sleep derangements and circadian dysfunction [2], the children in our study had better overall sleep quality.

Adult studies on this subject, including work by Grossman et al., have shown that adult patients consider vital signs, tests, noise, and medications to be top disruptors of sleep [5]. These factors significantly overlap with the top disruptors identified by this study. Other adult studies have specifically focused on environmental factors that disrupt sleep. For example, Dobing et al. found that the hospital environment is counterproductive to high-quality sleep due to excessive exposure to noise (59%) and frequent interruptions (30%) as the most commonly reported environmental disruptors to sleep [11]. Limited pediatric studies on inpatients have generated similar findings to our study. For example, Peirce et al. compared caregiver, physician, and nurse perspectives on the most disturbing sleep disruptors in the pediatric hospital setting and found that vital signs (50%), nurse/physician interruption (49%), and continuous pulse oximetry (38%) were the most disruptive to patient sleep [6].

The results of this study highlight that there are tangible changes hospitals can implement to improve patient sleep, especially a reduction in vital sign measurements in clinically stable patients. Lin et al. described a quality improvement project that resulted in a significant decrease in overnight vital sign measurements from 98% to 38% in low-risk pediatric patients with no adverse safety events; sleep outcomes were not measured in this study [12]. In a pilot study, Cook et al. showed improvement in pediatric sleep duration and decreased nighttime disruption with a reduction in overnight blood pressure measurements. Similarly, there were no adverse clinical outcomes with reduced overnight monitoring [13]. Future studies are needed to evaluate the effect of reduced vital signs on sleep outcomes.

It is notable that participants rated factors out of their personal control (vital signs, alarms, pain, and testing) as most disruptive, while factors that are within participant level of control (personal use of technology including cell phone and television) as least disruptive. Although participant bias may play a role, this finding suggests that when disruptors occur at unknown times or at times that are unclear or unexpected to patients, they cause more disruption than factors that patients and families can control directly. This finding suggests that interventions to improve sleep may include ways to help patients anticipate when some disruptions will occur (for example, vital signs and labs will not occur before a certain time or will occur at specified times) to give patients increased awareness and control over when their sleep may be disturbed.

There are several limitations of this study. This study focused on subjective sleep quality and timing. Further analyses using objective measures including actigraphy, a non-invasive measure of sleep and physical activity using a device worn on the wrist, would be helpful as additional data points and to correlate with participant and caregiver-reported findings. Although self-reported data have obvious limitations, when evaluating overall sleep health, the patient’s experience with sleep is an invaluable and important component to consider.

## Conclusions

In medically stable children, modifiable factors may be associated with improved patient perception of sleep in the hospital environment. Reductions in the frequency of vital sign measurements or scheduling vital signs in ways that are more sleep-friendly may improve patients’ perception of sleep quality. Simply giving advance notice of the anticipated timings of the potential disruptors overnight (such as advising patients when vital signs and laboratory studies will be obtained) may serve to decrease anxiety about loss of sleep and unintended arousals. Future studies to evaluate these factors and other approaches to improve the quantity and quality of sleep in hospitalized children are needed.
